# Economic Cognitions Among Older Adults: Parental Socialization Predicts Financial Planning for Retirement

**DOI:** 10.3389/fnagi.2017.00376

**Published:** 2017-11-21

**Authors:** Francisco Palaci, Irene Jiménez, Gabriela Topa

**Affiliations:** Department of Social and Organizational Psychology, Universidad Nacional de Educación a Distancia, Madrid, Spain

**Keywords:** aging process, financial cognitions, parental economic socialization, decision-making, financial management behavior

## Abstract

Drawing on the model on financial planning for retirement (FPR), the aim of this work is to explore how parental economic socialization both directly and indirectly affects FPR through the mediation of financial literacy, financial planning decisions and financial management. Data from a sample of 280 participants aged between 45 and 63 years were used. The results show that parental economic socialization directly and indirectly influences FPR. Moreover, parental economic behavior acts as a positive model for the development of financial literacy and skills and for decisions about FPR. All the variables increased the explained variance of FPR. Lastly, we discuss the process by which parental economic socialization is positively related to financial literacy and skills that impact on FPR, indicating some implications and future lines of research.

## Introduction

We currently live in a time of great economic changes, and the need to save for retirement is determined by the rise in the cost of living in the future and by the augmentation of the older population (Annoni and Weziak-Bialowolska, 2016; Budowski et al., 2016). Increased life expectancy and limited sustainability of public pension system underline that it will be very important to have sufficient economic resources to deal with retirement time (Lytle et al., 2015). Therefore, the appropriateness of individual savings and decisions about investments will be the key to successful aging in contemporary society (Teerawichitchainan and Knodel, 2015). Empirical investigations indicate that parents should take responsibility so their children will improve their levels of financial literacy develop adequate financial behaviors (Jorgensen and Savla, 2010), and learn good economic decision-making. Accordingly, recent research recommends exploring the interaction between parental socialization and financial outcomes under a mediation-based approach (Tang et al., 2015).

This study examines the influence of parental economic socialization on financial planning for retirement (hereafter FPR) and the mediating role of financial literacy, decisions about FPR and financial management, based on the model on FPR of Hershey et al. (2013). Despite the fact that the Hershey’s model provides answers to a wide range of present questions about FPR, further exploration of remote antecedents is needed in order to refine our understanding of FPR (Topa et al., submitted). This model states that FPR is determined by three dimensions: capacity, disposition, and opportunities to plan and save, as well as by the interaction that occurs between them.

The first dimension includes the abilities, knowledge and skills that contribute to differences in people’s cognitive and intellectual capacity to effectively plan and save for retirement. The second dimension includes the motivational forces and the psychological and emotional factors that determine the likelihood that an individual will begin planning and will sustain the activity over time. Finally, certain external influences, including environmental facilitators and constraints, influence effective financial tasking.

In the present study, financial literacy was considered as belonging to the capacity dimension, decisions about FPR are within disposition dimension, financial management belongs to the opportunity dimension and parental economic socialization is a distal antecedent of FPR because, as pointed out by some authors, financial lessons learned in childhood could have an impact 20 or 40 years later (Dan, 2004). Therefore, the main goal of this work is to determine the extent to which parental economic socialization accounts for individuals’ FPR by means of various mediator processes.

## Financial Planning for Retirement (FPR)

Savings have been and continue to be the main starting point to describe financial behavior and, normally, people accumulate their savings during their professional careers to have economic resources during retirement. Many recent studies have focused on FPR (among others, Love et al., 2008; Venti, 2011) because planning the financial aspect of retirement is increasingly important to ensure one’s financial security at this stage (McLaughlin, 2016; Whitley et al., 2016).

This planning consists of assessing future financial needs and saving to achieve economic well-being during retirement. Considering the studies that have investigated FPR, it has been observed that planning and saving for retirement is related to a feeling of financial security and to the acquisition of more wealth in comparison with those who do not plan economically (Ameriks et al., 2003; Ekici and Koydemir, 2016). In addition, the perception of social norms could affect the tendency to plan and save, as well as the impact of the person’s financial knowledge and skills (Hershey et al., 2013). Financial literacy has received the most attention concerning FPR and it has been related to saving for retirement (Hershey et al., 2007; Gutierrez and Hershey, 2013; Kiso and Hershey, 2016; Koposko et al., 2016).

## Parental Economic Socialization

During adolescence and later on during youth, individuals try out familiar behaviors and new behaviors in order to learn how to be adults (Campbell, 1969; Montoya-Castilla et al., 2016). In the economic context, this translates into a progressive attempt to individually resolve economic problems that arise in daily life. Economic psychology considers both the influence of economy and of the social and cultural environment; in the case of children and adolescents, their parents are the main environmental influence (Sonuga-Barke and Webley, 1993). In addition, adolescence is an important life stage in which economic socialization occurs, as specific attitudes toward saving develop. Recent research indicates that early experiences continue to be associated with attitudes, even in later life, and may not be completely overridden by adult experiences (Ward, 2013).

Previous studies showed that parental economic behavior had an impact on the children’s economic behavior (Feather, 1991). Subsequently, Otto (2009) studied parents’ role in the development of their children’s saving skills during adolescence, finding that their saving example influenced their children’s saving skills. In the same line, other works have found that parental economic socialization is related to children’s greater financial literacy in adolescence (Gutierrez and Hershey, 2014) and it also predicts saving behavior (Hira et al., 2009). Similar results were also found in older samples, which showed that parental economic socialization also influenced university students’ saving behaviors and economic planning (Thung et al., 2012).

To sum up, early influences had a large impact on saving behavior in later life, as Brown and Taylor (2016) stated. In this sense, it should not be forgotten that, as a result of the economic crisis that originated in 2008, people are more interested in improving their financial skills and are gradually taking on more personal responsibility to achieve financial well-being. On the basis of this literature, the present work proposes that:

- H1: Parental economic socialization will predict FPR.

## Financial Literacy

As stated in above, it seems reasonable to think that the relation between parental economic socialization and FPR is indirect and may be influenced by other not yet clearly established variables, partly because the transition from childhood to adulthood is understudied by economic psychology (Otto, 2009). Accordingly, one of the possible mediators is financial literacy. This consists of the skill to read, manage and analyze the personal finances that have an impact on one’s financial well-being (Nawaz, 2015). We know that receiving financial education can improve both financial behavior and literacy since empirical results in this regard have been found.

First, it was noted that taking finance courses improved the financial behavior of adults aged between and 25–55 years (Lyons et al., 2006). Second, with regard to FPR, various works have found that financial literacy is positively related to saving behavior, to investment performance (Nawaz, 2015), and to pension plan participation (Koposko et al., 2016). In addition, it was found that people with more financial literacy plan for their retirement to a greater extent (Koposko and Hershey, 2014). Finally, Drever et al. (2015) have recently proposed a theoretical model that relates economic socialization to responsible and efficient financial management through the development of literacy, specific cognitive skills, and personality characteristics that promote financial management in adulthood. Hence, the present study proposes:

- H2: The relation between parental economic socialization and FPR will be mediated by financial literacy.

## Decisions About FPR

The decision to prepare for retirement constitutes the prior stage to preparation for retirement *per se*, and specifically, economic preparation is one of the most important aspects to be planned. The decision to start preparing for retirement largely depends on socially accepted norms (Goodnow, 1997). Thus, people often begin to prepare for retirement when they notice that their coworkers or peers begin to prepare for it. In addition, various studies were conducted to discover the factors that influence the decision to prepare economically for retirement. First, it has been observed that the decision is influenced by the person’s vision of the future, such that people who live from day to day will find it difficult to decide to plan financially for retirement (Jacobs-Lawson and Hershey, 2005). In addition, having sufficient economic resources to save has also been associated with the decision to prepare financially for retirement (Hira et al., 2009). Furthermore, this decision also depends on people’s retirement goals, for example, if they intend to travel a lot at this stage, they are more likely to decide to plan and save for retirement (Schulz, 2002). To sum up, the lifespan planning and decision making would be influenced by personal experiences (Smeaton et al., 2017), as early financial learning (Koposko and Hershey, 2014). Therefore, in the present work, we propose that parental economic socialization will exert indirect influence on FPR through decisions about FPR.

- H3: The relation between parental economic socialization and FPR will be mediated by the decisions about FPR.

## Financial Management

Initially, Deacon and Firebaugh (1988) stated that financial management includes a series of behaviors aimed at planning, implementation, and assessment in the areas of cash, credit, investments, insurance and retirement. With regard to consequences of financial management, it has been observed that, when families use effective financial management, their economic welfare improves, whereas inadequate financial management can lead to negative social consequences at the long term (Choi, 2017). If the income of a family is inadequate to meet the financial obligations, the capacity to save will be affected (Garasky et al., 2008). It has also been observed that youth practice fewer basic financial skills, such as budgets, developing regular savings plans or planning for long-term needs (Birari and Patil, 2014).

Financial management involves a series of behaviors that differ in their frequency among individuals, for example, paying with credit cards tends to be more common than signing a pension plan. It has also been pointed out that repeated exposure to and practice of financial activities could help to develop financial literacy and skills in economic transactions (Jorgensen and Savla, 2010). Lastly, it was found that people who score high in financial management have higher levels of savings and lower levels of debt (Dew and Xiao, 2011). To sum up, as life course perspective stated (Elder, 1998); individuals are influenced by significant others and, at the same time, they could shape their lives by choosing certain activities to engage. Hence, parental economic influences could be combined with personal decision to plan and save. This may have important implications in FPR because, as noted, parental economic socialization has an impact on present and future economic behavior and general financial management is one of these behaviors.

- H4: The relation between parental economic socialization and FPR will be mediated by financial management.

**Figure 1:**
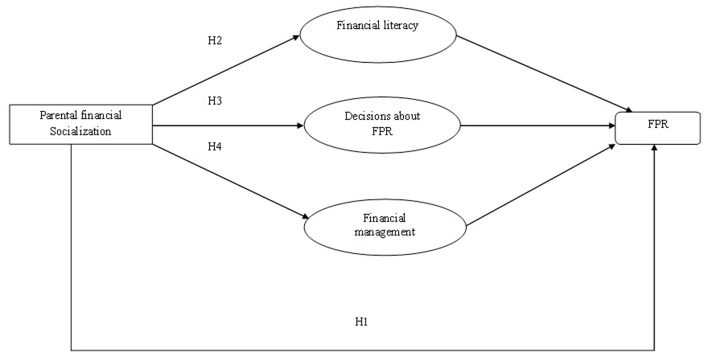
Hypothesized model for the study. Note: FPR, financial planning for retirement.

The hypothesized model for this study is displayed in Figure 1.

## Materials and Methods

### Participants

Following the “two-stage sampling” approach suggested by Van Solinge (2013), a specific pool of small and medium-sized enterprises was selected first, and the actual population of workers aged between 45 years and 63 years in the firms concerned were then invited to participate. This procedure allows us to avoid some shortcomings of the empirical studies on retirement. On the one hand, empirical studies are often criticized for relying on convenience samples affected by unknown selectivity issues. On the other hand, certain published studies take representative national samples so as to obtain generalizable results for the population of older adults as a whole. However, this involves further restrictions in order to whittle samples down to the limited number of participants actually transitioning to retirement.

Hence, our procedure entailed two steps. The research group contacted 10 firms to propose a broad study of human resources management. The eight firms that responded were then visited by the researchers to explain the criteria for the inclusion of participants (current employees over the age of 45 years). Only five organizations finally took part in the study. During May–July of 2017, 355 current employees aged 45 years or older received the questionnaire, a letter explaining the purpose of the study and the data collection procedure, and an envelope to return the survey directly to the Research group. Those employees who agreed to participate completed the questionnaires individually and outside of their workplaces. We eventually collected 302 completed questionnaires, yielding a response rate of 85%. Questionnaires missing more than 25% of the data were excluded, yielding a final sample of 280 Spanish participants.

The Ethical Committee of the three authors’ University Bio-ethical Committee of the National Distance Education University (UNED) approved the Project at May 2016. This study was carried out in accordance with the recommendations of Declaration of Helsinki revised in Fortaleza (World Medical Association [WMA], 2013), followed and approved by the Bio-ethical Committee of the National Distance Education University (UNED). All subjects gave written informed consent in accordance with the Declaration of Helsinki. The protocol was approved by the Bio-ethical Committee of the National Distance Education University (UNED). Confidentiality was maintaining by data being de-identified prior to data analysis by the deletion of identifiable material. Regarding gender, 59.3% of the sample was female (40.7% male). The participants’ mean age was 53.08 years (*SD* = 5.06). The most predominant professional categories were employee (62.5%) and intermediate manager (22.5%). Mean job tenure in the company was 19 years (*SD* = 10). Concerning education, 40.7% of the sample had university studies, and 30.7% up to high school. Moreover, 29.3% worked in the health setting, and 22.5% in the technological setting. Regarding job status, 89.3% worked full time, and 2.9% were part-time employed. With regard to retirement, 55% thought they could retire at age 65 years and 17% at 67 years.

### Measures

Demographic data, including the age, gender, level of training and education, number of dependants in the household, work setting, job tenure in the company, employment status, professional category and estimated retirement age were collected from the participants through a set of items included at the beginning of the questionnaire. Regarding the structure of the questionnaire, all the items of the following scales were mixed in a random order.

#### Parental Economic Socialization

We used a scale adapted to adults with eight items to measure parental economic socialization based on the “Parents as a guide” subscale included in the “Saving Attitude Scale” of Otto (2009) and the “Parental Socialization Scale” of Thung et al. (2012). Example items of this scale are: “I usually save because my parents taught me to do so when I was a child” or “My parents are proud of me because I am thrifty”. All the items were rated on a five-point response format ranging from 1 (strongly disagree) to 5 (strongly agree). This measure has shown high internal consistency in the past (*α* = 0.75; Thung et al., 2012) and the value of Cronbach’s alpha found in this study was 0.86.

#### Financial Literacy

We used a six-item scale to measure individuals’ general financial literacy (Hershey and Mowen, 2000; Jacobs-Lawson and Hershey, 2005). Example items of this scale are: “I know a lot about FPR” or “When I need to consult about finances I know exactly where to get the information”. All items are rated on a 5-point response format ranging from 1 (strongly disagree) to 5 (strongly agree). This measure has shown high levels of internal consistency in the past (*α* = 0.94; Jacobs-Lawson and Hershey, 2005) and the value of Cronbach’s alpha found in this study was 0.77.

#### Decisions About FPR

The decisions about FPR were measured by means of the Financial Preparation subscale included in the “Decision to Prepare for Retirement Scale” (Noone et al., 2010). The participants completed four items using a 5-point scale ranging from 1 (strongly disagree) to 5 (strongly agree). Examples of these items are: “In my financial situation, I think it’s too early to begin to think about when I retire” or “I shall worry about financial issues when I am closer to retirement”. This measure has shown high internal consistency in this study (*α* = 0.73).

#### Financial Management

We used the Financial Management Scale (Dew and Xiao, 2011), which consists of 15 items included in four subscales (savings and investments, cash management, credit, and insurance management). The instructions were: “Please indicate the frequency with which you have carried out the following activities in the past 6 months”. Examples of the items used are: “Paying all your bills on time” or “Buying bonds, shares, or funds”. All items used a 5-point response scale ranging from 1 (never) to 5 (always). This measure has shown high values of internal consistency in the past (*α* = 0.81; Dew and Xiao, 2011), and the value of the Cronbach’s alpha found in this study was 0.73.

#### FPR

To measure FPR, we used the Financial Planning for Retirement Scale (Stawski et al., 2007). Participants completed nine items using a 7-point scale ranging from 1 (strongly disagree) to 7 (strongly agree). The instructions required participants to answer items about the financial planning activities they had carried out in the past 12 months. Example items are: “I have made specific expenditure plans for the future” and “I have made voluntary contributions to a savings plan for retirement.” This measure has shown high values of internal consistency in the past (*α* = 0.87; Stawski et al., 2007), and the value of Cronbach’s alpha found in this study was 0.74.

## Results

### Descriptive Analyses and Correlations

The means, standard deviations and correlations of all the variables in this study are shown in Table 1. Related to control variables, both age and organizational tenure also correlated positively and significantly with FPR (both *r* = 0.15, *p* < 0.05), but the number of dependants and estimated age of retirement correlated negatively but non significantly with FPR. FPR was significantly and positively correlated with parental economic socialization (*r* = 0.14, *p* < 0.05), financial literacy (*r* = 0.25, *p* < 0.01), decisions about FPR (*r* = 0.19, *p* < 0.01), and financial management (*r* = 0.41, *p* < 0.01).

**Table 1:** Descriptive statistics and correlation matrix of variables (*N* = 280).

Variables	M	SD	1	2	3	4	5	6	7
**Control variables**
1. Age	53.08	5.06	−
2. Number of dependants	1.04	1.16	−0.34**	−
3. Organizational tenure	18.81	10.01	0.40**	−0.08	−
**Predictor variables**
4. Parental economic socialization	3.33	0.78	−0.02	0.12*	0.06	−
5. Financial literacy	2.84	0.73	−0.13*	0.19*	−0.05	0.27**	−
6. Decisions on FPR	3.26	0.81	0.18**	−0.01	0.02	0.12*	0.31**	−
7. Financial management	3.23	0.55	−0.10	0.08	−0.02	0.33**	0.44**	0.30**	−
**Criterion variable**
8. FPR	2.76	0.84	0.15*	−0.01	0.15*	0.14*	0.25**	0.19**	0.41**

Note: *p < 0.05; **p < 0.01.

With regard to the descriptive statistics of the target variables, the highest score was observed in parental economic socialization (*M* = 3.33, *SD* = 0.78), the lowest score in financial literacy (*M* = 2.84, *SD* = 0.73), and FPR obtained a mean of 2.76 (*SD* = 0.84).

### Hypothesis Testing

We analyzed the relation of parental economic socialization with FPR, both directly and considering the variables financial literacy, decisions about FPR and financial management as mediators. To test the first hypothesis of the study, linear regression analysis was conducted, entering the control variables in the first step (Model 1: age, number of dependants in the household and job tenure in the company), and parental economic socialization was entered in the second step (Model 2). The first step was nonsignificant, but in the second step, parental economic socialization predicted FPR positively and significantly (*β* = 0.14, *p* < 0.05), which provides support for Hypothesis 1. However, the values of *R*^2^ were not higher than 0.10.

To analyze the mediation hypotheses, we used the INDIRECT macro for SPSS elaborated by Preacher and Hayes (2008). The direct effect of parental economic socialization on FPR was nonsignificant in all of the three mediation cases: financial literacy (*c*’ = 0.08, *p* = 0.24), decisions about FPR (*c*’ = 0.12, *p* = 0.05), and financial management (*c*’ = −0.01, *p* = 0.92). Likewise, with a 95% confidence level, the confidence intervals (CI) did not include the value 0 (CI financial literacy [0.03, 0.13], decisions about FPR [0.002, 0.07] and financial management [0.10, 0.24]), so the indirect effect was significant. Therefore, the results confirmed that financial literacy, decisions about FPR and financial management mediated the relation between parental economic socialization and FPR, which confirms Hypotheses 2–4 (Tables 2–5).

**Table 2:** Linear regression analysis on FPR.

Predictor variables	FPR	
	Model 1 *β*^a^	Model 2 *β*^a^
Age	0.12	0.12
Number of dependants	0.04	0.02
Organizational tenure	0.11	0.10
Parental economic socialization		0.14*
R^2^	0.03	0.05
F	2.45*	3.09*
ΔR^2^	0.03	0.02
ΔF	2.45*	5.46*

Note: ^a^Standardized regression coefficients; FPR, Financial Planning for Retirement (N = 280). *p < 0.05.

**Table 3:** Regression results for testing mediation of financial literacy in the relationships between parental economic socialization and FPR.

	Coeficient	SE	*t*	axb	EE	95% CI
Total effect	0.15*	0.06	2.34
Direct effect	0.07	0.07	1.16
H2: Parental economic socialization → Financial literacy → FPR				0.07	0.02	[0.03, 0.13]

Note: N = 280; SE, Standard error; CI, confidence interval; unstandardized regression coefficients are reported. Bootstrap sample size = 10.000; *p < 0.05.

**Table 4:** Regression results for testing mediation of decisions on FPR in the relationships between parental economic socialization and FPR.

	Coeficient	SE	*t*	axb	EE	95% CI
Total effect	0.15*	0.06	2.34
Direct effect	0.12	0.06	1.97
H3: Parental economic socialization → Decisions on FPR → FPR				0.02	0.01	[0.002, 0.07]

Note: N = 280; SE, Standard error; CI, confidence interval; unstandardized regression coefficients are reported. Bootstrap sample size = 10.000; *p < 0.05.

**Table 5:** Regression results for testing mediation of financial management in the relationships between parental economic socialization and FPR.

	Coeficient	SE	*t*	axb	EE	95% CI
Total effect	0.15*	0.06	2.34
Direct effect	−0.01	0.06	−0.10
H4: Parental economic socialization → Financial management → FPR				0.16	0.03	[0.10, 0.24]

Note: N = 280; SE, Standard error; CI, confidence interval; unstandardized regression coefficients are reported. Bootstrap sample size = 10.000; *p < 0.05.

## Discussion

In the environment of accelerated economic changes currently undergone by society, people must increase their responsibility for their economic stability in the last stages of their lives. For this purpose, they should improve their decisions and financial skills because these will allow them to perform more responsible financial behavior. The role of the parents as socializing agents is very important for the origin and development of these decisions and skills. In addition, we know that parental financial teaching influences the efficacy of financial behaviors (Shim et al., 2015).

The main goal of this work was to determine whether parental economic socialization was positively related to FPR through other variables, thus providing support for the hypotheses proposed from the model of Hershey et al. (2013). First, parental economic socialization was positively and significantly related to FPR through financial literacy, coinciding with the findings of other recent studies (Shim et al., 2015). Moreover, our study empirically verifies some of the theoretical formulations presented by Drever et al. (2015), who establish a model of relations between economic socialization and responsible financial management through a set of mediator variables such as skills, literacy and personality traits.

Second, it is interesting that some works did not find a relationship between financial literacy and FPR or else they found a very weak one (Tang et al., 2015). Likewise, in another work, no differences were observed between the financial behavior of people who took a financial course and that of those who did not (Mandell and Klein, 2009). In any case, there seems to be empirical evidence that parental socialization plays a role in facilitating and promoting saving behavior, even a long time later. Accordingly, our findings agree with other works that indicate strong links between parental influence and responsible financial behavior, confirming these relations in longitudinal investigations with more than 20 years of follow-up of the participants, such as study of Tang et al. (2015) or, with a shorter interval, in the work of Shim et al. (2015), or Trzcińska and Goszczyńska (2015).

Third, parental economic socialization is positively and significantly related to FPR through decisions about FPR (H3). This coincides with the idea that what we learn from our parents and what they teach us about economic issues and savings has an impact on the decision to plan the financial aspect of retirement, and this decision is the first step of planning for retirement. Thus, Koposko and Hershey (2014) find that early parental influences affect future time perspective and retirement goal clarity, which are the antecedents of the tendency to plan and save. On another hand, it has been noted that this decision could also be affected by the person’s estimation about his or her future retirement needs and, despite the importance of these decisions, it is remarkable that there are so few empirical works on the topic (Topa et al., submitted). In any event, very promising pathways of future research are opening because parental socialization practices could also be influencing through the perceptions of financial self-efficacy that children develop, which have been shown to be predictors of positive retirement outcomes in other studies (Montford and Goldsmith, 2016).

Fourth, it should not be forgotten that various works indicate that the family is not the only influence in financial decisions, but instead they are also influenced by comparisons with peers (Koposko et al., 2016), or by socialization coming from other sources. Accordingly, particularly important is the work of Dávila et al. (2016), which compares peer influence and parental influence in Spanish children, concluding that the former have more impact than the latter on classmates’ materialism. These results should be related to our findings on the family in future research. In the same vein, also extending the period of familiy influences taken into account could be useful, along with developing other approaches as generational to expand future findings (Lim et al., 2015).

Fifth, parental economic socialization was positively and significantly related to FPR through financial management, supporting the last hypothesis of this investigation (H4). In a similar vein, it has been observed that financial management is related to people’s financial attitudes (Mien and Thao, 2015), and economic socialization plays an important role in these attitudes. Although the socialization of parents on FPR is fundamental, as the study underlines and concludes, in a context of unemployment and job insecurity and a consumer society such as the current one, it is interesting to not forget that working conditions in adulthood, political and social disaffection that could also influence financial decisions, as an anonymous reviewer suggested.

With regard to the theoretical implications of the present investigation, to our knowledge, there are no precedents that have examined parental socialization from the perspective of a full model of FPR, although there are works about responsible financial behavior in general (Shim et al., 2015; Tang et al., 2015), or retirement attitudes (Macewen et al., 1995). Despite the fact that empirical studies on FPR have been conducted from other theoretical perspectives, such as the theory of planned behavior (Thung et al., 2012), the model used herein takes into account various dimensions that affect FPR, and allows developing more solid hypotheses. Thus, taking the data as a whole, empirical support was obtained for the indirect relation between parental economic socialization and FPR.

### Limitations

As this is a cross-sectional study, we did not analyze the changes that occur in the variables as time elapses, and this must be taken into account when interpreting the findings of this research because longitudinal works are often found in economic psychology (Shim et al., 2015; Tang et al., 2015). In any event, these works frequently focus on the general population of children or adolescents, and not on the study of adults’ FPR with these designs. Despite the cross-sectional nature of the data, we highlight that most investigations on this topic use the same type of design, due to the difficulty of collecting data about parental socialization in childhood and adolescence in order to assess its influence on adults’ FPR 40 years later.

In addition, the representativeness of the Spanish sample used hinders generalization of the results, as we know that depending on the culture and the pension systems (Khan et al., 2016); some variables may be more significant than others in predicting FPR (Mansor et al., 2015). Notwithstanding, diverse studies of parental economic socialization carried out with very diverse samples such as Europe (Otto, 2009), the United States (Koposko and Hershey, 2014), Poland (Trzcińska and Goszczyńska, 2015) or Malasia (Thung et al., 2012) have shown consistent relations between parental influences and saving behaviors. On another hand, there are various measures of financial management, and some of them focus more on saving management, investment management, or cash management. Here, we chose a global measure, and this may affect the results (Resende and Zeidan, 2015). With regard to the procedure used to collect the data, self-report measures may lead to common variance error. In this sense, some recent works indicate the need for standardized measurement instruments to assess financial literacy, as the different measures can lead to results that may not be comparable (Schmeiser and Seligman, 2013). In relation to these methodological issues, some authors also point out that the position of key information in documents and advertisements concerning financial products can affect the accessibility of the information and, as a result, influence decision-making (Foster et al., 2015).

### Future Lines of Research

This work coincides with other studies in which the model of behavior that the parents teach their children has a positive impact on the development of financial behaviors and literacy but it would be interesting to explore whether parental economic socialization has negative repercussions on the children’s economic decisions and behaviors. Nevertheless, future works could examine the weight of different types of economic socialization (parents, teachers, peers, etc.) on FPR because, although parents are the first source of influence, other models of financial behavior may influence throughout adolescence and adulthood, and it would be appropriate to study the interaction between all of them, as outlined above.

In the same vein, future research would consider that parental economic socialization should interact with emotions or other individual features (Rodríguez et al., 2015; Yu and Chen, 2016) or personality characteristics (Topa and Herrador-Alcaide, 2016) in order to affect retirement planning.

Moreover, despite the fact that our participants were 60% women, it seems that there is no influence of gender from the professional profile of respondents. Due to the fact that the influence of being female would be greater according to the differences on socio-economic status, as professional levels and salary, future research should take into account a different other profiles of lower professional women, along as different sectors or work (Haratsis et al., 2015).

In the socialization of parents it would be interesting to provide a gender perspective and to analyze, for future studies, whether the father’s influence is greater in that he usually has better working conditions (higher wages to equal work than women) and is usually the “bread winner or head of household” and decide more on family economic issues. Or, on the contrary, it is already mothers (with their double role as housewives and paid workers) who are increasingly influencing this. Due to gender differences, perhaps mothers tend to be less charged and therefore less influential on these issues, as an anonymous reviewer suggested.

Additionally, for future research, a qualitative sample could be used based on some in-depth interviews or discussion groups for children/youth or adults. These techniques would allow us to obtain opinions on the values, attitudes and expectations (desires or reality) of these groups of people on important subjects that are discussed here: savings/spending, consumption and consumerism, need for preparation and training for retirement economic security) from childhood to improve intergenerational relations, need for savings for reasons of leisure, health, future care, etc. Especially due to the fact that the family or the State can no longer cover this service anymore (Van der Heijden et al., 2016).

In addition, the role of the level of income in the relationship between financial literacy and FPR should be of examined because authors like Pahnke and Honekamp (2010) found that financial literacy was positively related to FPR only in the case of high economic income. Lastly, we wonder whether parents’ teachings about economic material are direct—through their communication—, indirect—by means of observation—, or whether both of them interact and if so, how, in order to establish the most efficacious parental socialization practices in this matter, as proposed from developmental psychology (Shim et al., 2015).

### Practical Implications

This work reveals the importance of parental economic teachings, both at the level of theoretical and practical knowledge, in the individuals’ saving decisions for FPR. In a similar vein, continuing with the development of financial education, schools should include classes or courses to improve financial skills and knowledge that enable students to interpret the information they receive from television, internet, banks, etc.

Likewise, for the adult population, programs of credit counseling could be created that highlight the benefits of long-term saving for financial security. Thus, instruction in economics and saving would be more complete and continued lifelong, as it is important to properly deal with possible economic changes that may arise at the family level (e.g., occupational dismissal, retirement) or at the population level (e.g., economic crisis, changes in the system of pensions; Han et al., 2015). As recent empirical research stated, the relationship between financial hardship and impaired well-being is strong (Annink et al., 2016). Moreover, counseling interventions should be expanded, in order to include novel findings regarding the use contemplation in the context of financial management. As an experimental study showed (Harkin, 2017), promting participants to contemplate their debt and expenditures would improve financial decision-making, by reducing the likelihood of avoid debt-related information and improve estimates of expenditures.

To sum up, this work supports earlier studies that show that parental financial behavior impacts on children’s FPR, and it is a positive model for the development of financial literacy and skills and for decisions about FPR. Thereby, the findings support the idea that FPR can and must start at youth because we have the tools for this purpose, and delaying FPR could reduce the likelihood of success. Hence, this initial learning, complemented with others developed during adulthood, is of great relevance for FPR.

## Author Contributions

FP, IJ and GT made substantial contributions to the conception or design of the work; drafting the work or revising it critically for important intellectual content; final approval of the version to be published; agreement to be accountable for all aspects of the work in ensuring that questions related to the accuracy or integrity of any part of the work are appropriately investigated and resolved.

## Conflict of Interest Statement

The authors declare that the research was conducted in the absence of any commercial or financial relationships that could be construed as a potential conflict of interest.
